# Associations of mobile source air pollution during the first year of life with childhood pneumonia, bronchiolitis, and otitis media

**DOI:** 10.1097/EE9.0000000000000007

**Published:** 2018-02-23

**Authors:** Caitlin M. Kennedy, Audrey Flak Pennington, Lyndsey A. Darrow, Mitchel Klein, Xinxin Zhai, Josephine T. Bates, Armistead G. Russell, Craig Hansen, Paige E. Tolbert, Matthew J. Strickland

**Affiliations:** a Department of Environmental Health, Rollins School of Public Health, Emory University, Atlanta, GA; b Oak Ridge Institute for Science and Education, Oak Ridge, TN; c School of Community Health Sciences, University of Nevada Reno, Reno, NV; d School of Civil and Environmental Engineering, Georgia Institute of Technology, Atlanta, GA; e Kaiser Permanente Georgia Center for Clinical and Outcomes Research, Atlanta, GA; and f Centre for Traumatic Stress Studies, University of Adelaide, Adelaide, Australia.

**Keywords:** Pneumonia, Bronchiolitis, Otitis media, Air pollution, Traffic

## Abstract

**Background:**

Exposure to air pollution from motor vehicles in early life may increase susceptibility to common pediatric infections.

**Methods:**

We estimated associations between residential exposure to primary fine particulate matter, nitrogen oxides, and carbon monoxide from traffic during the first year of life and incident pneumonia, bronchiolitis, and otitis media events by age 2 years in 22,441 children from the Kaiser Air Pollution and Pediatric Asthma Study, a retrospective birth cohort of children born during 2000–2010 and insured by Kaiser Permanente Georgia. Time to first clinical diagnosis of each outcome was defined using medical records. Exposure to traffic pollutants was based on observation-calibrated estimates from A Research LINE-source dispersion model for near surface releases and child residential histories. Associations were modeled using Cox proportional hazards models, with exposure as a continuous linear variable, a natural-log transformed continuous variable, and categorized by quintiles.

**Results:**

During follow-up, 2,181 children were diagnosed with pneumonia, 5,533 with bronchiolitis, and 14,374 with otitis media. We observed positive associations between early-life traffic exposures and all three outcomes; confidence intervals were widest for pneumonia as it was the least common outcome. For example, adjusted hazard ratios for a 1-unit increase in nitrogen oxides on the natural log scale (a 2.7-fold increase) were 1.19 (95% CI = 1.12, 1.27) for bronchiolitis, 1.17 (1.12, 1.22) for otitis media, and 1.08 (0.97, 1.20) for pneumonia.

**Conclusions:**

Our results provide evidence for modest, positive associations between exposure to traffic emissions and common pediatric infections during early childhood.

## Abstract 2

Supplemental Digital Content is available in the text.

## Introduction

Pneumonia, bronchiolitis, and otitis media are common pediatric infections with a large economic burden. Pneumonia is the second leading cause of infant mortality globally and is the leading cause of pediatric hospitalization in the United States, with associated medical costs amounting to almost $1 billion.^1,2^ In 2002, an estimated 149,000 children under the age of two in the United States were hospitalized for bronchiolitis, resulting in $543 million in direct medical costs and $1.4 billion in costs associated with hospitalizations.^3^ An estimated 8.7 million children are diagnosed with otitis media annually in the United States, with an associated cost of $2.88 billion in health care utilization.^4^ Understanding environmental risk factors for these common pediatric outcomes will help prioritize public health interventions in geographic areas of greatest need.

Due to their relatively small size, greater time spent outdoors, more active behaviors, greater inhalation rates, and incomplete development of the respiratory and immune systems, children are more vulnerable to ambient air pollutants than adults.^5,6^ Epidemiologic evidence suggests that both short-term and long-term exposure to traffic-related air pollution may be a risk factor for respiratory infections and related comorbidities in infants and children, with potential lasting effects on respiratory health later in life.^6–13^ Secondary pollutants, including ozone and particulate matter (PM), were found to be significantly associated with increased risk for bronchitis, pneumonia, and otitis media in a study of Georgia pediatric emergency department visits during 2002–2008.^14^ Jedrychowski et al^15^ reported a dose–response relationship between recurrent bronchiolitis and pneumonia infections with PM_2.5_ at the child’s prenatal residence in Krakow, Poland. Moreover, modest and positive associations between nitrogen oxides (NO_x_), PM_2.5_, and PM_10_ with otitis media among children have been reported in several studies^16–20^ but not others.^12,21^ A meta-analysis of 10 European birth cohorts found elevated and significant associations between NO_2_ and PM_10_ with pneumonia in early childhood,^18^ but most cohorts did not assess outcomes in infancy. Proximity studies have suggested a 6% and 14% higher risk of bronchiolitis infection and hospitalization, respectively, for infants living in dense traffic areas,^22,23^ and others have found similar results.^24^ Interventions to reduce traffic emissions in large cities can have a major impact on decreasing pediatric respiratory diagnoses and hospitalizations as suggested by a 20-year study in California.^25^

In this article, we report associations between average residential fine PM_2.5_, carbon monoxide (CO), and NO_x_ concentrations from traffic during the first year of life and childhood pneumonia, otitis media, and bronchiolitis clinical encounters by age two in a cohort of children insured by the Kaiser Permanente Georgia Health Maintenance Organization in Atlanta, Georgia. Although the association of traffic-related air pollutants on the development of pediatric asthma^13,26^ and other developmental outcomes^27,28^ is relatively well-established, there is less evidence for associations with infectious outcomes, and our study contributes to this evidence base. Further, there are important between study differences in traffic exposure models that may be a source of heterogeneity in association estimates across studies, and the authors of a recent review called for further refinement of such models.^29^ Although most previous studies have used land use regression,^29^ here we use a newly developed fine-scale traffic dispersion model that is calibrated to measurements from stationary air pollution monitors.^30^ Finally, African American children are not well represented in the previous literature, and a contribution of our study is to provide estimates from a racially diverse pediatric population wherein 35% of children were identified as African American.

## Methods

### Ambient air quality model

We modeled hourly concentrations of primary PM_2.5_ (μg/m^3^), CO (ppm), and NO_x_ (ppm) contributed by mobile sources for 2002–2011 in metropolitan Atlanta at 250-m resolution using A Research LINE-source dispersion model for near surface releases (RLINE).^31^ This model is designed to estimate air quality emissions from traffic in the direct vicinity of the roadway by numerically integrating point source emissions while also accounting for local meteorological conditions that affect dispersion patterns. Model inputs included emissions data for roadway segments based on 2010 traffic data from the Atlanta Regional Commission’s Atlanta Roadside Emissions Exposure Study and surface meteorology data for 2002-2010 from AERMET, the meteorological processors of AERMOD (The American Meteorological Society/Environmental Protection Agency Regulatory Model).^32,33^

We created annual averages from the hourly estimates, and these averages were used in the epidemiologic analyses. The averages were calibrated using observational data from stationary air pollution monitors to adjust for overestimation of spatial gradients. Estimates of NO_x_ and CO were calibrated directly to observations because an estimated 73% and 88% of these pollutants, respectively, are contributed by mobile sources.^30^ A smaller proportion of PM_2.5_ is contributed by mobile sources, so primary PM_2.5_ was calibrated to source apportionment estimates based on monitoring data that were created using a chemical mass balance model with gas constraints.^34^ Annual average pollutant concentrations were created for years 2002–2011. The annual emissions during this time decreased by a factor of 1.8, 1.5, and 2.0 for PM_2.5_, CO, and NO_x_, respectively.^30^ Because the spatial characteristics of the study area (number/location of highways, traffic density, etc.) did not change meaningfully between 2000 and 2002, we assigned the year 2002 estimates to the study years prior to 2002, that is, 2000 and 2001.^30^ RLINE does not include mechanisms forming secondary PM_2.5_, so only the associations with the primary portion of the PM_2.5_ are assessed here. Further details about the creation of these mobile-source air pollution estimates are available.^30^

### KAPPA cohort data

The Kaiser Air Pollution and Pediatric Asthma Study (KAPPA) is a retrospective, medical records–based birth cohort of all children born between 2000 and 2010 to mothers in metropolitan Atlanta, Georgia, who were insured by Kaiser Permanente Georgia (KPGA) Health Maintenance Organization (HMO) for at least the first year of life. There were 24,608 children in the KAPPA study and 22,441 were included in this analysis. Children were excluded if they were not enrolled in KPGA at day 29 of life (the start of the outcome period of interest; n = 489); were diagnosed with pneumonia, bronchiolitis, or otitis media in the first 28 days of life (n = 223; to exclude common infections during the neonatal period); had no residential history information in the first year of life (n = 721); or had one or more residence during the exposure period outside the region for which pollutant concentrations were available (n = 734; since exposure classification was based on residence). The study was approved by the institutional review boards of both Emory University and KPGA.

The three health outcomes examined in this study were childhood pneumonia (International Classification of Diseases, 9th revision (ICD-9) codes 480-486), otitis media (ICD-9 codes 382.XX), and acute bronchitis and bronchiolitis (ICD-9 codes 466.XX). Because 80% of the events in the acute bronchitis and bronchiolitis outcome group were bronchiolitis (ICD-9 codes 466.1X), we refer to this outcome henceforth as “bronchiolitis.” For each outcome, we followed children from day 29 of life (to exclude neonatal infections) until time of the first diagnosis, censorship (e.g., they ceased to be insured by the HMO), or the child’s second birthday. Ambient concentrations of primary PM_2.5_, CO, and NO_x_ from traffic were assigned to each child based on residential location. We calculated separate estimates for each outcome using residences between the child’s birth date and date of diagnosis of the outcome of interest or their first birthday (whichever came first). When a child moved during the exposure period, we calculated a time-weighted average of the estimated concentrations at each location.

### Description of covariates

We adjusted for neighborhood socioeconomic status (SES) and city region, as well as several covariates available from medical records: child race, child sex, maternal asthma, maternal education, maternal prenatal smoking, birth year, and maternal age. These covariates were prioritized based on strength of the evidence in the literature, as well as data availability. Because the RLINE estimates are for annual averages, there is no seasonality in exposure concentrations and, hence, no confounding by season. Neighborhood SES was characterized at the census block group level by demographic clusters created by the Georgia Department of Public Health.^35^ These clusters were created using 2010 US Census data on 25 variables related to age, income, family structure, housing, education attainment, and employment. Neighborhood SES was determined for each child based on residence at birth. City region described the location of the child’s residence in Atlanta: inside metropolitan Atlanta (defined as inside the I-285 highway that surrounds the city), less than or equal to 16 km outside I-285, and more than 16 km outside I-285. Categorizations of other covariates were child race (white, black, other, unknown), maternal asthma status (no, yes, missing), maternal education (less than 12th grade, high school or equivalent, at least some college, missing), maternal smoking during pregnancy from birth certificate data (no, yes, missing), birth year indicator variables (2000–2010), maternal age dichotomized at the mean, and child sex (male, female).

### Statistical Modeling

We used Cox proportional hazards (PH) regression to estimate associations between first year of life exposure to primary PM_2.5_, CO, and NO_x_ from traffic emissions and time to first diagnosis of pneumonia, bronchiolitis, or otitis media (up until age 2 years). The PH assumption was evaluated using Kaplan–Meier log–log curves, goodness of fit tests using Schoenfeld residual *P* values, and extended Cox models to test each variable’s interaction with survival time. Variables satisfying the PH assumption according to (at least) two of these three approaches in univariate models were deemed to satisfy the PH assumption. Stratified Cox models were used to adjust for variables that did not satisfy the PH assumption. As expected, the pollutant exposure distributions were right skewed, which motivated an examination of modeling exposures as continuous linear variables (scaled to 1 μg/m^3^ PM_2.5_, 0.02 ppm NO_x_, and 1 ppm CO), as natural log-transformed continuous variables, and by quintiles. Data were analyzed using SAS 9.4 (Cary, NC) allowing for nonindependence due to sibling clustering via the robust sandwich estimator implemented by the “covs(aggregate)” statement in PROC PHREG.

## Results

**Figure 1.**
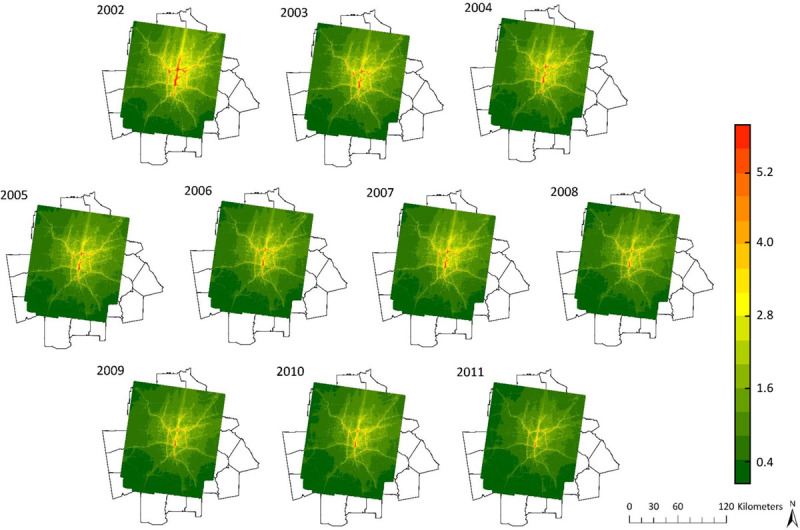
2002–2011 primary PM_2.5_ (µg/m^3^) concentrations contributed by mobile sources.

Annual average estimates for primary PM_2.5_, NO_x_, and CO from traffic are shown in Figure 1, eFigure 1; http://links.lww.com/EE/A2, and eFigure 2; http://links.lww.com/EE/A2. Descriptive statistics on first year of life air pollution exposures from traffic are shown in Table 1. Exposures were highly correlated (pairwise Spearman *r*_*s*_ between 0.97 and 0.99) because the three pollutants were all modeled using the RLINE method and because the concentrations of all three pollutants decreased over the course of the study period.

**Table 1:**
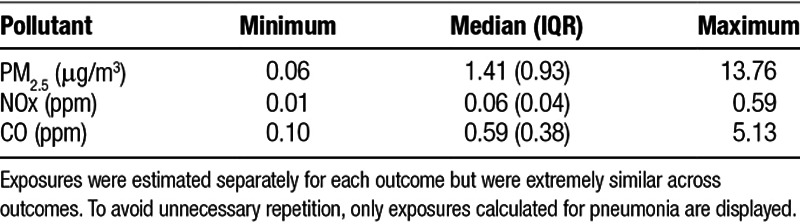
First year of life exposure to primary PM_2.5_, NOx, and CO from traffic (N = 22,441)

The 22,441 children in the cohort came from 18,640 different families. Follow-up through age 2 years was 78.0% (N = 17,502). Cohort characteristics are shown in Table 2. The cohort was racially diverse, with 39.5% of children classified as white, 34.9% as black, and the remaining classified as unknown or other race. The majority of children were born to mothers who attended at least some college and lived in neighborhoods classified as high SES. Out of the three outcomes examined by the second birthday, otitis media was the most common: 14,374 children (64.1%) were diagnosed with otitis media; 5,533 children (24.7%) were diagnosed with bronchiolitis; and 2,181 children (9.7%) were diagnosed with pneumonia. Males were more likely than females to be diagnosed with a respiratory or ear infection by age 2 years. Examining differences by race, black children were more likely to be diagnosed with pneumonia, and white children were more likely to be diagnosed with bronchiolitis or otitis media. Seasonal variation in diagnosis was observed, with the highest proportion of diagnoses occurring in winter for all outcomes (38% of pneumonia cases, 44% of bronchiolitis cases, and 34% of otitis media cases). Collectively, 18.2% of children in the cohort changed residences at least once during the first year of life.

**Table 2:**
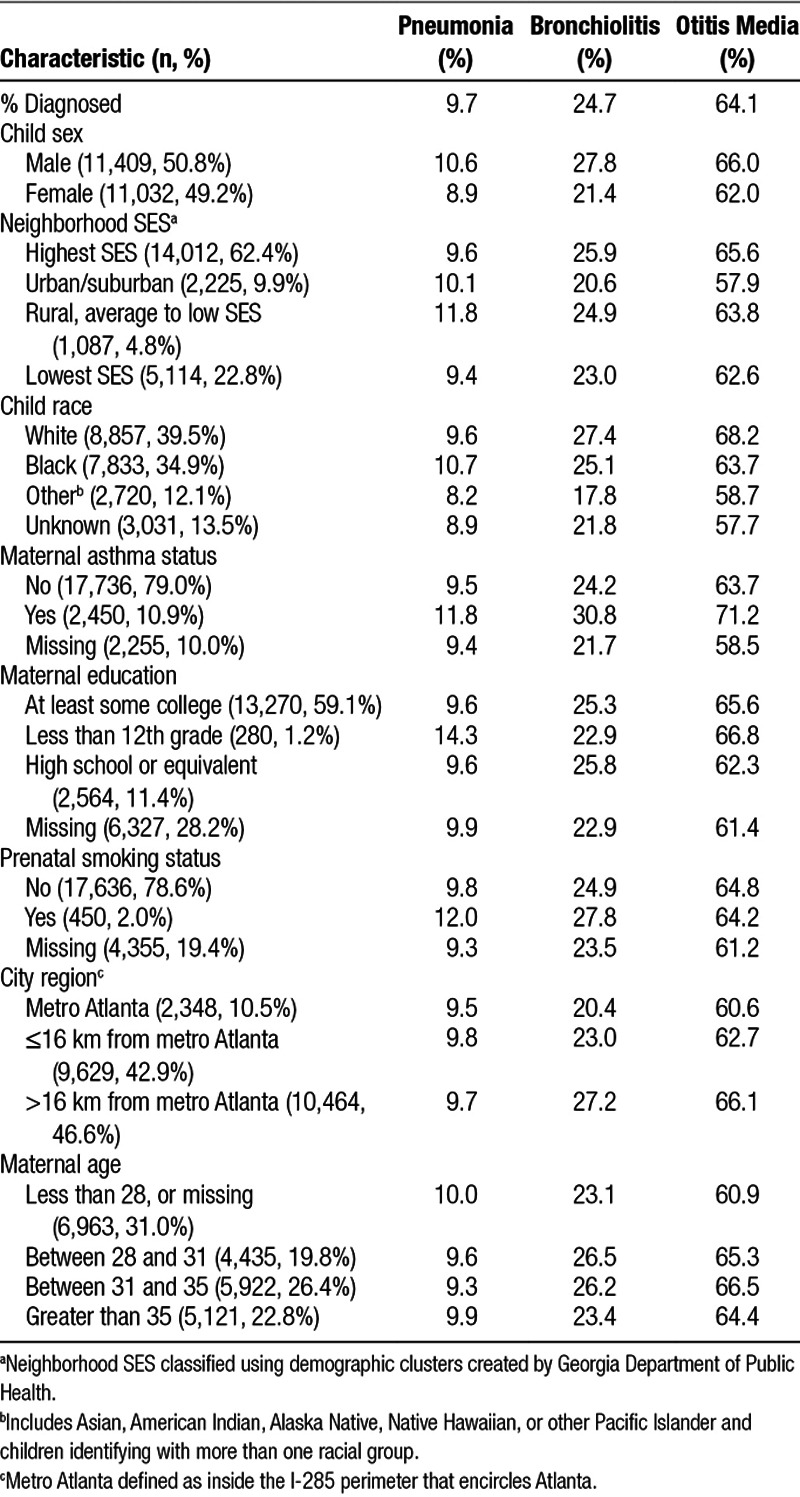
Descriptive statistics for children born between 2000 and 2010 and enrolled in Kaiser Permanente in the Atlanta, Georgia, metropolitan area (n = 22,441)

Neighborhood SES violated the proportional hazards assumption for both bronchiolitis and otitis media, child race violated the PH assumption for bronchiolitis, and city region violated the PH assumption for otitis media. No variables violated the PH assumption for pneumonia. We therefore implemented stratified Cox models for the bronchiolitis and otitis media analyses.

Although the magnitude of the hazard ratios varied, overall conclusions were consistent from Cox models when exposure was modeled as a continuous linear variable (eTable 1; http://links.lww.com/EE/A2) and as a natural log-transformed continuous variable (Table 3). For a log increase in exposure, unadjusted hazard ratios for all pollutants with pneumonia, bronchiolitis, and otitis media ranged from 0.95 to 1.05 with confidence intervals including the null (HR=1.0) in all but one instance (Table 3). The association estimates were elevated after statistical adjustment for covariates. In the adjusted models for bronchiolitis, the hazard ratios ranged from 1.16 (95% CI = 1.08, 1.25) for a 2.7-fold increase in CO (a 1-unit increase on the natural log scale) to 1.23 (95% CI = 1.15, 1.32) for a 2.7-fold increase in PM_2.5_. Adjusted hazard ratios were similar for otitis media. The adjusted HRs for pneumonia were also positive, but 95% confidence intervals were wider because pneumonia was the least common outcome. For example, the adjusted HR for pneumonia for a 1-unit increase in log-transformed PM_2.5_ was 1.08 (95% CI = 0.97, 1.20). Sex-specific association estimates are also shown in Table 3. Associations tended to be of larger magnitude for boys than for girls, with bronchiolitis showing the largest differences between the sex-specific HR estimates.

**Table 3:**
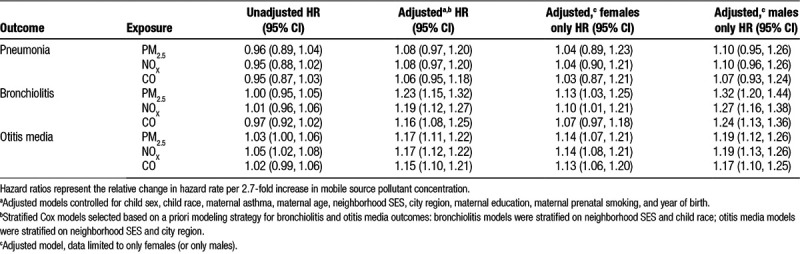
Hazard ratios per natural log increase in primary PM_2.5_, NOx, and CO from traffic and child outcomes by age 2 years (n = 22,441)

**Figure 2.**
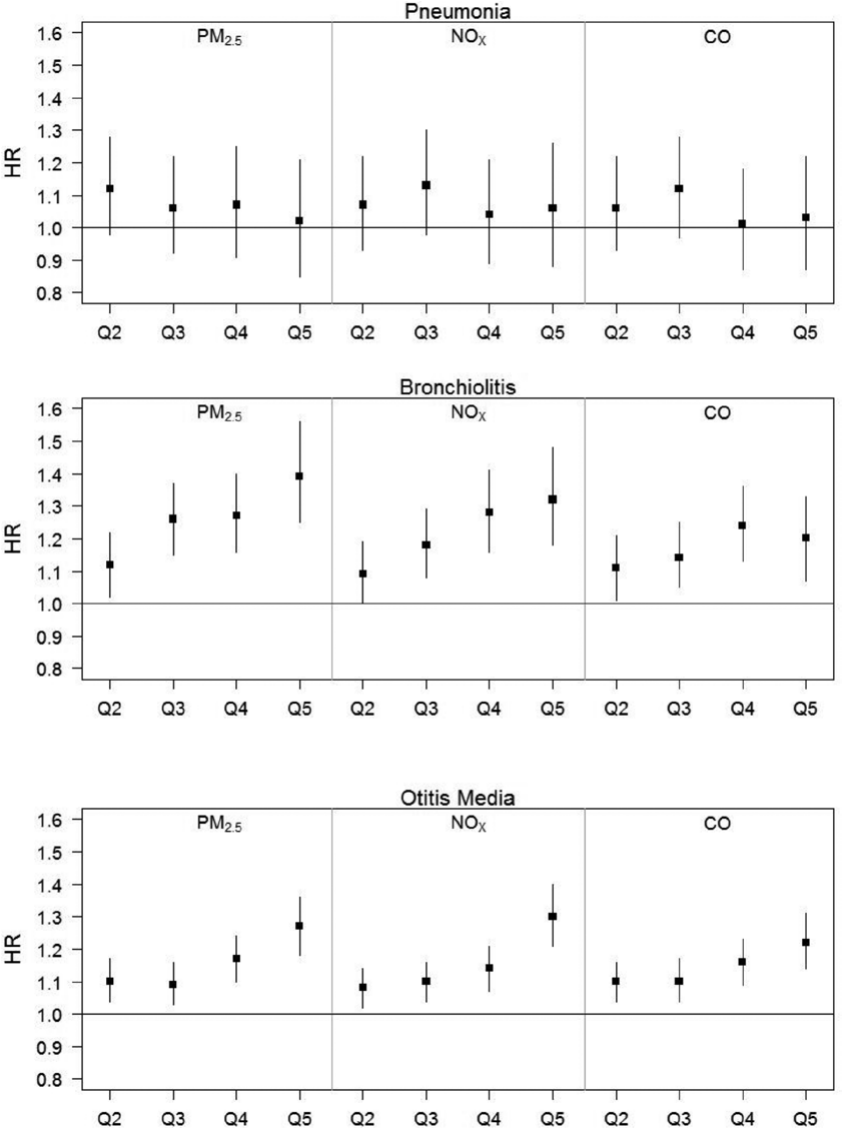
Adjusted hazard ratios and 95% confidence intervals per quintile of primary PM_2.5_, NOx, and CO from traffic and pneumonia, bronchiolitis, and otitis media by age 2 years (using quintile 1 as the reference group). Numeric results for this figure are available in eTable 2; http://links.lww.com/EE/A2.

When exposure was modeled using quintiles, we observed a general trend where the association estimates between primary PM_2.5_, NO_x_, and CO from traffic and bronchiolitis and otitis media by age 2 years tended to increase as the exposure quintiles increased (Figure 2). This pattern was not evident for pneumonia. For bronchiolitis and otitis media, visual inspection of the shape of the exposure–response relationship across quintiles suggested that the HRs increased more rapidly for bronchiolitis than for otitis media.

## Discussion

These results provide evidence for modest, positive associations between exposure to traffic emissions and bronchiolitis and otitis media diagnoses in the first 2 years of life. The estimated associations with pneumonia were positive but less elevated, and the confidence intervals were wider. Owing to the strong correlation that exists between pollutants, associations for a given health outcome tended to be similar across pollutant species, and we were unable to isolate their individual impacts. Consequently, we view our results as being indicative of an association between early childhood infections and the “mixture” of traffic-related air pollutants. We are less confident in drawing conclusions about the associations with the individual pollutants because of the high between-pollutant correlations and the inability to create multipollutant regression models. Similar issues with high correlation pertain to the comparison of results across various exposure windows, for example, first year of life exposures and prenatal exposures were highly correlated (Spearman *r*_*s*_ > 0.9 for all pollutants). These high correlations precluded investigation of the association of traffic-related pollutants during one exposure period while controlling for the levels of the pollutants during other periods. In a recent meta-analysis of traffic-related air pollutants and asthma incidence, the authors mention investigation of various exposure windows as an area for future research.^26^ To support such an investigation, future studies will need either to be very large or to have substantial variation in exposure across windows.

Our study considers the associations of three surrogates of early life ambient air pollution exposures from traffic on three common pediatric outcomes among a southern metropolitan cohort of insured children. Children and infants are especially vulnerable to air pollution because their lungs and immune systems are still developing and do not reach full biological potential until approximately 6 years.^6^ Air pollution exposures during this critical stage can overwhelm the body’s natural defense mechanisms and lead to lasting effects on respiratory health.^6^ PM is a primary component of diesel exhaust, which can stimulate reactions among T cells and induce allergic inflammatory response and induction of immune defense mechanisms.^36^ High doses of traffic-related aerosols may induce oxidative stress and mitochondrial damage and increase pulmonary retention of particles.^36^ Exposure to ambient air pollution during infancy and childhood is associated with increased hospitalizations and emergency room visits,^22–24^ adverse developmental outcomes,^27,28^ and increases in infant mortality.^37^

Misclassification of residential location in the KAPPA study is likely a small concern because the Kaiser Permanente Georgia HMO retains information on previous addresses, which enabled us to create time-weighted air pollution metrics. Although there is imprecision in the date of address change, simulations performed using KAPPA data to investigate the consequences of exposure measurement error due to residential mobility suggest that in this cohort, this source of error likely causes only a small (2–10%) bias towards the null.^38^

The calibrated RLINE estimates were shown in Zhai et al^30^ to have good accuracy and precision; the calibration reduced normalized mean bias for all pollutants when compared to raw RLINE estimates (29% to 0.3% for PM_2.5_, 22% to −1% for CO, and 303% to 43% for NO_x_). A limitation of this model is that traffic emissions data were only available for 2010. Moreover, this analysis assumes constant population density, vehicle consumption, and land use over the course of the study period in the metropolitan Atlanta area. Although we used the network of regulatory monitors to calibrate the pollutant concentrations for the earlier years of the study, our air quality model would not have captured meaningful variability in traffic dynamics or intensity in the early years of our study period (2002–2009). However, we do not expect large changes in the spatial distribution of emissions to have occurred during or prior to this period as there were no major changes in freeways or major highways (calibration partially captures temporal changes, including impacts from the recession and emission controls).

The outcomes of interest in this study were defined using ICD-9 codes from clinical diagnosis instead of parental self-report, which lessens the potential for recall bias and outcome misclassification. This is a strength of our study; much of the prior literature has relied either on parental self-report^7,8,21^ or on emergency department visits and hospitalizations,^12,16,20,22,23,39^ which do not capture the less-severe morbidities that are treated in pediatric care offices. Even with these clinical records, however, it is very likely that some children had one or more of the outcomes but were never seen by a clinician, particularly due to the similar symptoms that these conditions can have to cold or flu. As such, the true incidence of these pediatric conditions is likely somewhat higher than what was captured by the medical records.

We used Cox proportional hazards regression to analyze the data, wherein we considered children to be at risk for illness until the date of their illness diagnosis or censorship (which occurred when a child reached age 2 years or when they were no longer insured by Kaiser Permanente Georgia). Thus, this analysis only considers the first occurrence of each outcome and does not consider recurrent infection or severity of outcome. Due to skewed exposure distributions, we modeled exposure as an untransformed continuous variable, transformed by the natural log, and by quintile to allow for a potential linear or nonlinear relationship between exposure and outcome. Results from all modeling techniques led to similar conclusions.

The KAPPA cohort is not a random sample of Atlanta children, as membership was limited to children with health insurance through Kaiser Permanente Georgia HMO. Only a small proportion of Atlanta children are insured through Kaiser Permanente. Broadly, the KAPPA children tended to be of higher SES than the general population, with 59% of mothers having at least some college education and 62% of children residing in neighborhoods classified as having the highest of the four SES categories. One advantage of a high socioeconomic status cohort is that risk of bias from residual confounding by socioeconomic factors might be lower than if the cohort consisted of a more diverse sample of Atlanta children. We do not know if the estimated associations with traffic pollutants would have been different had our cohort consisted primarily of children from lower socioeconomic status households. However, it is probable that certain factors that could plausibly lessen the exposure associations in this cohort, for example, air conditioning use and good nutrition, were more prevalent among KAPPA cohort children. Some potential confounders, such as breastfeeding or daycare attendance, were not available on the Kaiser Permanente medical records, and residual confounding by these factors is a possibility. Selection bias due to HMO enrollment attrition is also possible, although there were no differences in average air pollutant exposures between children who remained enrolled and those who left the cohort.

In this large urban birth cohort, we observed positive associations between concentrations of primary PM_2.5_, NO_x_, and CO from traffic emissions and childhood bronchiolitis and otitis media diagnoses. Associations with pneumonia were also positive, although the effect estimates were of relatively smaller magnitude, and the confidence intervals were wider. Our study, which integrates calibrated RLINE outputs with a rich data set of pediatric clinical encounters from Kaiser Permanente Georgia, provides further evidence regarding the associations between low-level residential traffic pollution during infancy and pediatric respiratory disease.

## Conflicts of interest statement

The authors declares that they have no conflicts of interest with regard to the content of this report.

This work was supported by grant R834799 from the U.S. Environmental Protection Agency, grants R03HD084884-01 and T32HD052460 from the National Institutes of Health, and grant 5T03OH008609 from the U.S. Centers for Disease Control and Prevention. This publication’s contents are solely the responsibility of the grantee and do not necessarily represent the official view of the US EPA. Further US EPA does not endorse the purchase of any commercial products or services mentioned in the publication.

Kaiser Permanente data are not available for redistribution. Parties interested in pursuing a data use agreement should contact the Center for Health Research Southeast Division of the Kaiser Foundation Health Plan, Inc.

## Supplementary Material
