# Supplementary material for: Associations of mobile source air pollution during the first year of life with childhood pneumonia, bronchiolitis, and otitis media
Source: Environ Epidemiol. 2018 Feb 23;2(1):e007. doi: 10.1097/EE9.0000000000000007 (PMC6133251; doi:10.1097/EE9.0000000000000007)
Supplement: Supplementary file 1 [file ee9-2-e007-s001.pdf]

eFigure 1. 2002-2011 NO_X (ppb) concentrations contributed by primary mobile sources

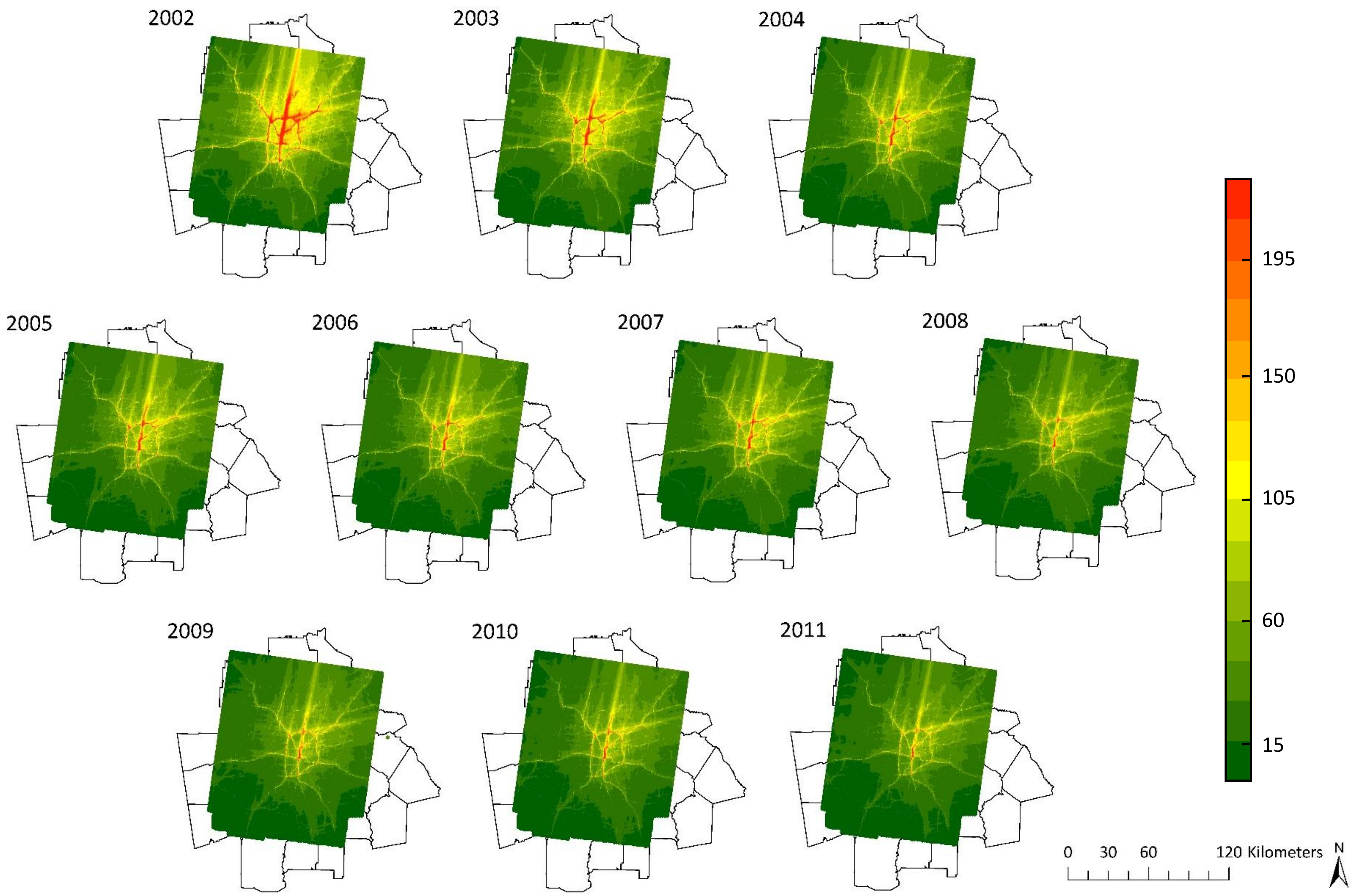

eFigure 2. 2002-2011 CO (ppm) concentrations contributed by primary mobile sources

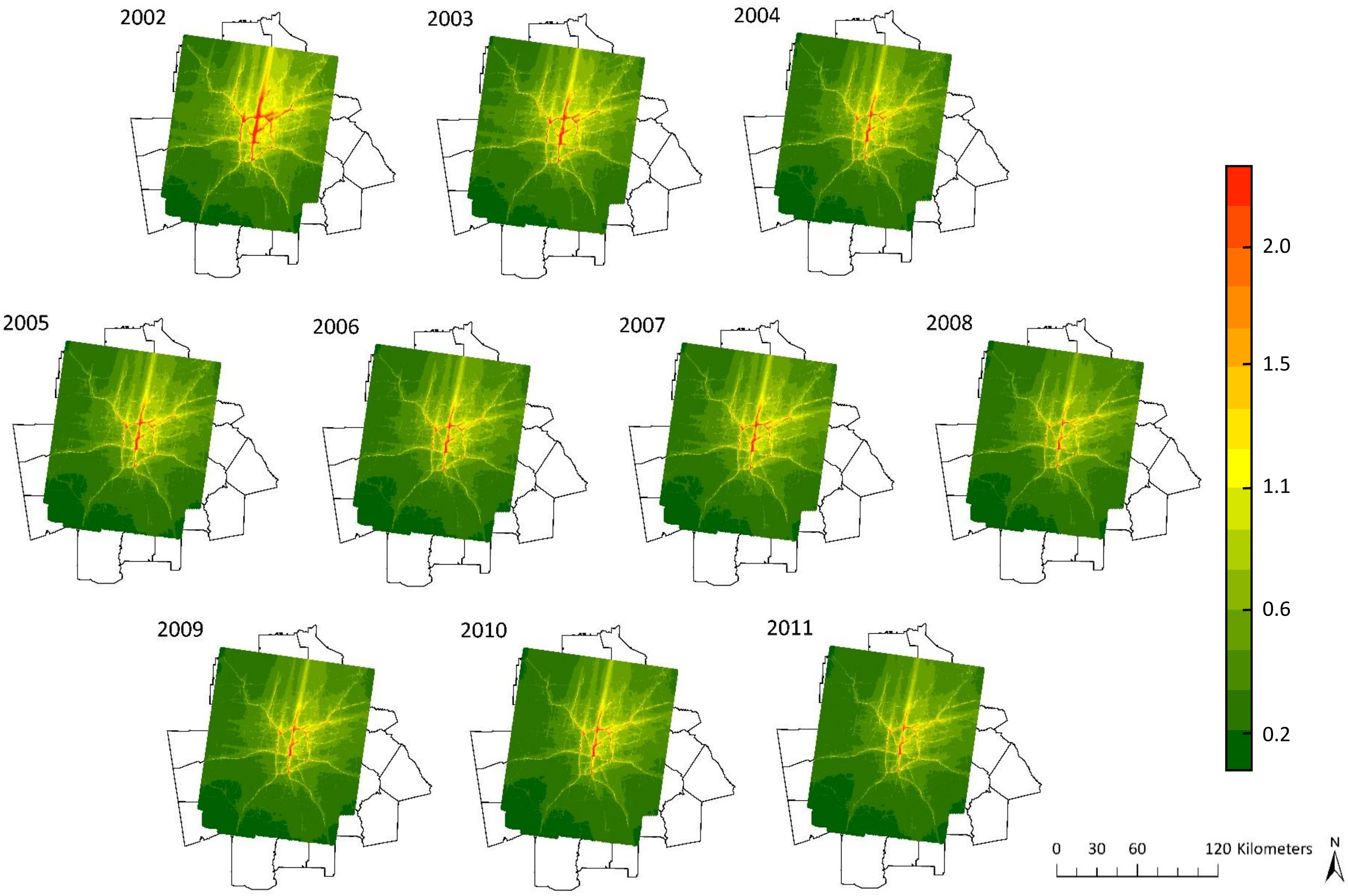

eTable 1. Hazard ratios for mobile source air pollutant concentrations and pneumonia, bronchiolitis, and otitis media by age two (n=22,441)

**Outcome**	**Exposure**	**Unadjusted HR (95% CI)**	**Adjusted[1,2] HR (95% CI)**
Pneumonia	$PM_{2.5}$ per 1 μg/m^3	0.96 (0.91, 1.02)	1.02 (0.96, 1.09)
	NO_X per 20 ppb	0.98 (0.96, 1.01)	1.01 (0.98, 1.04)
	CO per 1 ppm	0.92 (0.81, 1.05)	1.04 (0.89, 1.22)
Bronchiolitis	$PM_{2.5}$ per 1 μg/m^3	0.98 (0.95, 1.01)	1.10 (1.05, 1.14)
	NO_X per 20 ppb	1.00 (0.98, 1.01)	1.03 (1.02, 1.05)
	CO per 1 ppm	0.91 (0.84, 0.98)	1.13 (1.03, 1.24)
Otitis media	$PM_{2.5}$ per 1 μg/m^3	1.00 (0.98, 1.02)	1.07 (1.04, 1.10)
	NO_X per 20 ppb	1.01 (1.00, 1.02)	1.03 (1.02, 1.05)
	CO per 1 ppm	0.99 (0.95, 1.04)	1.14 (1.08, 1.21)

[1] Adjusted models controlled for child sex, child race, maternal asthma, maternal age, neighborhood SES, city region, maternal education, maternal prenatal smoking, and year of birth.

[2] Stratified models selected based on a priori modeling strategy for bronchiolitis and otitis media outcomes: bronchiolitis models were stratified on neighborhood SES and child race; otitis media models were stratified on neighborhood SES and city region.

eTable 2. Adjusted hazard ratios by quintile of mobile source air pollutant concentrations (relative to the lowest exposure quintile) and pneumonia, bronchiolitis, and otitis media by age 2

Outcome	Exposure	Pollutant Quintile			
		Quintile 2 HR (95% CI)	Quintile 3 HR (95% CI)	Quintile 4 HR (95% CI)	Quintile 5 HR (95% CI)
Pneumonia	$PM_{2.5}$	1.12 (0.98, 1.28)	1.06 (0.92, 1.22)	1.07 (0.91, 1.25)	1.02 (0.85, 1.21)
	NO_X	1.07 (0.93, 1.22)	1.13 (0.98, 1.30)	1.04 (0.89, 1.21)	1.06 (0.88, 1.26)
	CO	1.06 (0.93, 1.22)	1.12 (0.97, 1.28)	1.01 (0.87, 1.18)	1.03 (0.87, 1.22)
Bronchiolitis	$PM_{2.5}$	1.12 (1.02, 1.22)	1.26 (1.15, 1.37)	1.27 (1.16, 1.40)	1.39 (1.25, 1.56)
	NO_X	1.09 (1.00, 1.19)	1.18 (1.08, 1.29)	1.28 (1.16, 1.41)	1.32 (1.18, 1.48)
	CO	1.11 (1.01, 1.21)	1.14 (1.05, 1.25)	1.24 (1.13, 1.36)	1.20 (1.07, 1.33)
Otitis Media	$PM_{2.5}$	1.10 (1.04, 1.17)	1.09 (1.03, 1.16)	1.17 (1.10, 1.24)	1.27 (1.18, 1.36)
	NO_X	1.08 (1.02, 1.14)	1.10 (1.04, 1.16)	1.14 (1.07, 1.21)	1.30 (1.21, 1.40)
	CO	1.10 (1.04, 1.16)	1.10 (1.04, 1.17)	1.16 (1.09, 1.23)	1.22 (1.14, 1.31)

Using quintile 1 as the reference group. Models control for child sex, child race, maternal asthma, maternal age, neighborhood SES, city region, maternal education, maternal prenatal smoking, and year of birth. Stratified models selected based on a priori modeling strategy for bronchiolitis and otitis media outcomes: bronchiolitis models were stratified on neighborhood SES and child race; otitis media models were stratified on neighborhood SES and city region.

Quintile cut points: $PM_{2.5}$ ($\mu g/m^3$): <0.91, 0.91-<1.25, 1.25-<1.59, 1.59-<2.08, ≥2.08; CO (ppm): <0.38, 0.38-<0.52, 0.52-<0.66, 0.66-<0.86, ≥0.86; NO_X (ppb): <34.22, 34.22-<48.38, 48.38-<63.51, 63.51-<86.11, ≥86.11
